# Bioconversion of distillers’ grains hydrolysates to advanced biofuels by an *Escherichia coli* co-culture

**DOI:** 10.1186/s12934-017-0804-8

**Published:** 2017-11-09

**Authors:** Fang Liu, Weihua Wu, Mary B. Tran-Gyamfi, James D. Jaryenneh, Xun Zhuang, Ryan W. Davis

**Affiliations:** 1 Department of Biomass Science & Conversion Technologies, Sandia National Laboratories, Livermore, CA 94550 USA; 2 Department of Systems Biology, Sandia National Laboratories, Livermore, CA 94550 USA

**Keywords:** Distillers’ grains with solubles (DGS), Microbial co-culture, One-pot bioconversion, Fusel alcohol, Algae hydrolysate

## Abstract

**Background:**

First generation bioethanol production utilizes the starch fraction of maize, which accounts for approximately 60% of the ash-free dry weight of the grain. Scale-up of this technology for fuels applications has resulted in a massive supply of distillers’ grains with solubles (DGS) coproduct, which is rich in cellulosic polysaccharides and protein. It was surmised that DGS would be rapidly adopted for animal feed applications, however, this has not been observed based on inconsistency of the product stream and other logistics-related risks, especially toxigenic contaminants. Therefore, efficient valorization of DGS for production of petroleum displacing products will significantly improve the techno-economic feasibility and net energy return of the established starch bioethanol process. In this study, we demonstrate ‘one-pot’ bioconversion of the protein and carbohydrate fractions of a DGS hydrolysate into C4 and C5 fusel alcohols through development of a microbial consortium incorporating two engineered *Escherichia coli* biocatalyst strains.

**Results:**

The carbohydrate conversion strain *E. coli* BLF2 was constructed from the wild type *E. coli* strain B and showed improved capability to produce fusel alcohols from hexose and pentose sugars. Up to 12 g/L fusel alcohols was produced from glucose or xylose synthetic medium by *E. coli* BLF2. The second strain, *E. coli* AY3, was dedicated for utilization of proteins in the hydrolysates to produce mixed C4 and C5 alcohols. To maximize conversion yield by the co-culture, the inoculation ratio between the two strains was optimized. The co-culture with an inoculation ratio of 1:1.5 of *E. coli* BLF2 and AY3 achieved the highest total fusel alcohol titer of up to 10.3 g/L from DGS hydrolysates. The engineered *E. coli* co-culture system was shown to be similarly applicable for biofuel production from other biomass sources, including algae hydrolysates. Furthermore, the co-culture population dynamics revealed by quantitative PCR analysis indicated that despite the growth rate difference between the two strains, co-culturing didn’t compromise the growth of each strain. The q-PCR analysis also demonstrated that fermentation with an appropriate initial inoculation ratio of the two strains was important to achieve a balanced co-culture population which resulted in higher total fuel titer.

**Conclusions:**

The efficient conversion of DGS hydrolysates into fusel alcohols will significantly improve the feasibility of the first generation bioethanol process. The integrated carbohydrate and protein conversion platform developed here is applicable for the bioconversion of a variety of biomass feedstocks rich in sugars and proteins.

**Electronic supplementary material:**

The online version of this article (10.1186/s12934-017-0804-8) contains supplementary material, which is available to authorized users.

## Background

Global bioethanol production reached 25.7 billion gallons in 2015 [1], with further increase in annual production projected. During the ethanol refining process, starch in the grain flour is converted into ethanol and the remainder of the grain components, such as proteins, lipids and fibers comprise a residual coproduct, commonly known as distillers’ grains with solubles (DGS) [2]. It is estimated that in the dry milling process, the utilization of a bushel of corn (56 lb) results in 2.8 gallon of ethanol and 18 lb of DGS [3]. In 2015, 40 million metric tons of DGS were produced from US ethanol biorefineries [4]. DGS is considered as a rich source of cellulosic polysaccharides (52–57%), protein (27–31%), oil (10–12%) and other nutrients [2] and has long been marketed as a ruminant feed adjunct. However, due to the variability in nutrient content and digestibility issues as well as other concerns such as mycotoxins, antibiotic residues, sulphur content and the risk of introducing bacterial pathogens [5], acceptance of DGS in the feed industry has been limited. Alternatively, because of its vast supply and sugar and protein content, DGS is a potentially promising biomass source for upgrading to valuable fuel products using bioconversion strategies that are compatible with the established starch ethanol process. Therefore, efficient valorization of DGS to produce value-added products would significantly improve the techno-economic feasibility of the established starch bioethanol process.

Recent advances in synthetic biology, metabolic engineering, and systems biology, have enabled rapid progress in developing microbial factories [6–8] and novel enzyme cascade systems [9–11] for the synthesis of biofuels and other chemicals. When considering a microbial system for biomass conversion, although there are successful examples in developing ‘superbugs’ capable of multiple functions, engineering a single microbe to simultaneously perform multiple tasks is still quite challenging and bioenergetically costly under most situations, especially when utilizing complex substrates or performing complicated biosynthesis. Alternatively, well-designed microbial consortia involving two or more microbes that can take advantage of individual microbes and their interactions to realize synergistic division of labor and more efficient utilization of biochemical substrates, and therefore exhibit better properties than monocultures, should provide enhanced productivity, stability or metabolic efficiency [12–14].

**Fig. 1:**
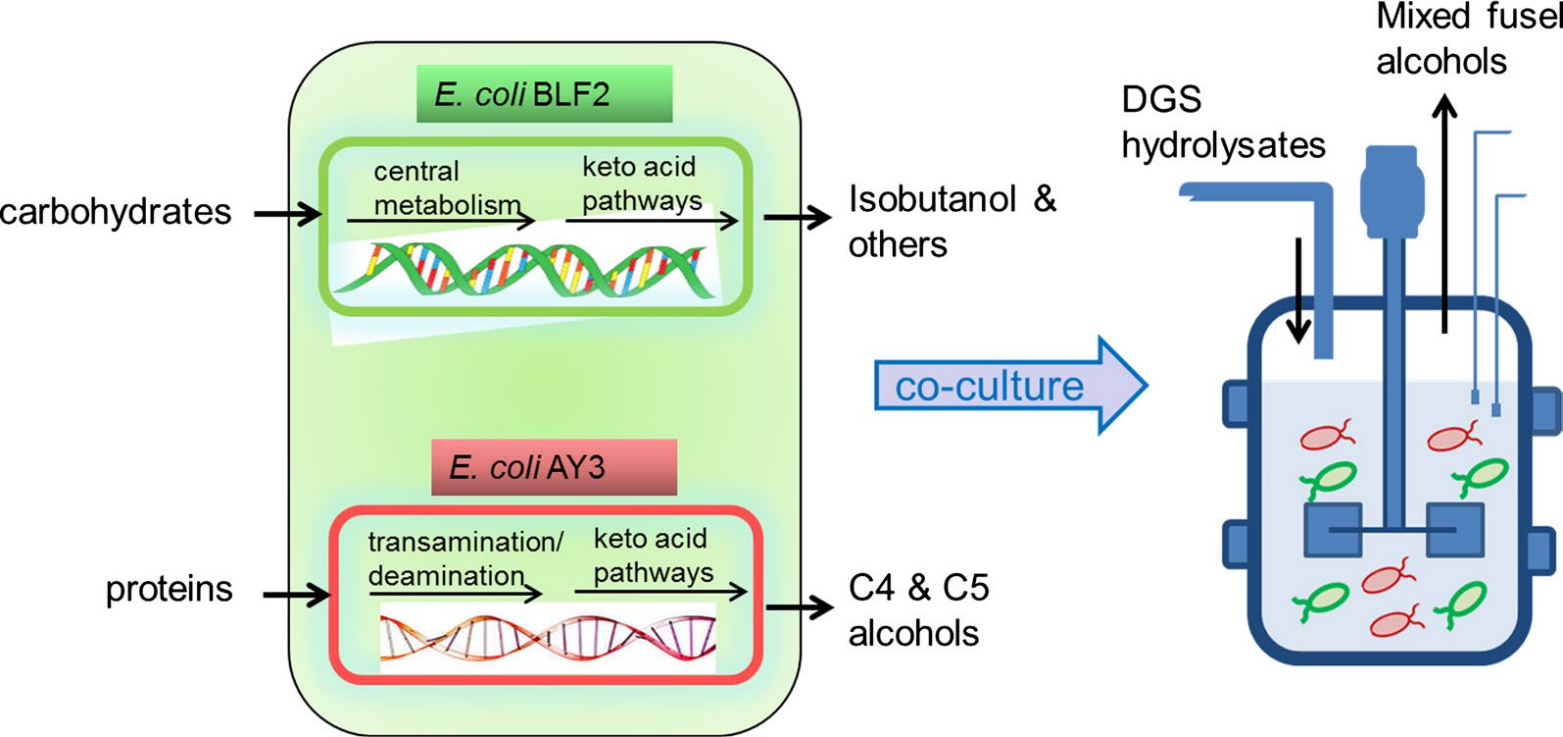
Schematic of one-pot bioconversion of DGS hydrolysates into advanced biofuels by an engineered *E. coli* co-culture

Ethanol has been successfully produced as a fuel product from the sugar fractions in pretreated DGS hydrolysates by an engineered yeast [15]. Recent studies suggest that fusel alcohols, primarily isobutanol (C4) and isopentanols (C5), which contain higher carbon content than ethanol (C2) have improved physical properties and higher energy densities than ethanol and are therefore considered as compatible, and in some cases, superior gasoline blending agents than ethanol [16]. Here, we developed an *E. coli* co-culture that is capable of simultaneously converting sugars as well as proteins in the DGS hydrolysates to produce fusel alcohols. In the engineered co-culture system, one *E. coli* strain was constructed for efficient conversion of hexose and pentose sugars in the DGS hydrolysates to isobutanol and other fusel alcohols. The second *E. coli* strain was modified for efficient utilization of the proteins in the DGS hydrolysates to produce mixed C4 and C5 alcohols. By co-culturing these two *E. coli* strains, we demonstrate ‘one-pot’ bioconversion of the protein and carbohydrate fractions of DGS hydrolysate into advanced biofuels (Fig. 1). Furthermore, a quantitative PCR-based cell quantification method was developed to enumerate the dynamics of each individual bacterial population in the co-culture.

## Methods

### Strains and plasmids

*Escherichia coli* strain B (ATCC 11303) was purchased from ATCC. *E. coli* AY3 was previously developed in our lab [17]. The mutant strain *E. coli* B01 with single deleted gene Δ*ldh*::cam^+^ was constructed using the technique of one-step disruption of chromosomal genes [18] using primers 5′-GGATGGCGATACTCTGCCATCCGTAATTTTTACTCCACTTCCTGCCAGTTTGTGTAGGCTGGAGCTGCTTC-3′ and 5′-CGCTATTCTAGTTTGTGATATTTTTTCGCCACCACAAGGAGTGGAAAATGTGACATGGGAATTAGCCATGGTCC-3′ from *E. coli* B strain.

To construct pLF101, part of the *ilvD* gene was PCR amplified using primers 5′-GTAAAAAATATGTTCCGCGCAGGTCC-3′ and 5′-TTTATTTGATGCCTCTAGCACGCGTACGCGTTTAACCCCCCAGTTTC-3′ using pYX90 [19] (generously provided by Professor James C. Liao from University of California, Los Angeles) as the template. The rrnB T1 terminator was amplified using primers 5′-ACGCGTGCTAGAGGCATCAAATAAAAC-3′ and 5′-AGTGAGCGAGGAAGCGGAATATATC-3′ using pYX90 as the template. Then the two fragments were assembled with *Sbf*I and *Avr*II digested pYX90 to achieve pLF101-alaS-ilvC-ilvD using In-Fusion^®^ HD Cloning Kit (Clontech, CA) following the manufacture’s protocol. To construct pLF102, part of the Amp^R^ gene and pLacO1 region was amplified using primers 5′-GCAAAAAAGCGGTTAGCTCCTTCG-3′ and 5′-CTCCTACTGTATACATGGTATATCTCCTTGTCGACAATGAATTCGGTCAGTGCGTCCTG-3′. The PCR fragment was assembled with *Pvu*I and *Sal*I digested pYX97 [19] (generously provided by Professor James C. Liao from University of California, Los Angeles) using In-Fusion^®^ HD Cloning Kit (Clontech, CA). The DNA sequences of the constructs were confirmed by DNA sequencing. The plasmids pLF101 and pLF102 were co-transformed into the *E. coli* strain B01, which generated the production strain *E. coli* BLF2.

### Medium and culture conditions

M9 medium containing 40 g/L glucose or 40 g/L xylose or 20 g/L glucose and 20 g/L xylose, 5 g/L yeast extract, 100 µg/mL ampicillin, 34 µg/mL chloramphenicol, and 25 µg/mL spectinomycin, and 1000th dilution of Trace Metal Mix A5 (Sigma-Aldrich, MO) was used for cell growth. The cells were inoculated in 3 mL medium in the test tube and incubated at 37 °C overnight at 250 rpm. 200 µL of the overnight culture was inoculated into 20 mL fresh medium in the shake flask and incubated at 37 °C, 250 rpm. 1 mM isopropyl-β-d-thio-galactoside (IPTG) was added when OD_600_ reached 0.8. Then the culture was grown at 30 °C and 250 rpm for 2 days. Samples were collected at regular time intervals for further analysis.

### Biomass pretreatment and fermentation

The distillers’ grains samples (35% solids) were provided by Aemetis, Inc. (Cupertino, CA) and pretreated following the protocols from the National Renewable Energy Laboratories. Briefly, DGS were mixed with 4% dilute sulfuric acid to a final concentration of 8.5% (w/v) solid. Then the mixture was incubated in the 90 °C water bath for 5 h and neutralized with Ca(OH)_2_ solids until pH reached 6.5. The resulting slurry was subsequently hydrolyzed with 1.5 mg/mL Pronase (Sigma-Aldrich, MO) following the manufacture’s protocol. After enzymatic digestion, the slurry was centrifuged and the supernatant was sterilized by filtration through the 0.22 µm PTFE membrane (Fisher Scientific, CA). *Nannochloropsis* sp. algae samples were pretreated similarly but incubated with 10% sulfuric acid. The resulting hydrolysates were used directly as the medium for cell growth and fusel alcohol production with no additional supplements.

*Escherichia coli* BLF2 cells were cultivated in 10 mL LB medium and grown at 37 °C, 250 rpm. The overnight culture was centrifuged at 4000 rpm for 10 min and the cell pellets were collected and washed with corresponding hydrolysates twice and resuspended in 1 mL hydrolysates. 0.8 mL of the mixture was inoculated into 20 mL DGS or algae hydrolysates supplemented with 100 µg/mL ampicillin, 34 µg/mL chloramphenicol, and 25 µg/mL spectinomycin. The culture was incubated at 37 °C, 250 rpm and induced with 1 mM IPTG when the OD_600_ reached 0.8. The flasks were cap-sealed and cultured for another 48 h at 30 °C, 250 rpm for fusel alcohol production. Samples were taken at the beginning and end of the fermentation for further analysis.

For co-culture fermentation, *E. coli* strains AY3 and BLF2 were cultivated in 10 mL LB medium separately. The overnight culture was centrifuged and the final cell pellets were individually re-suspended into 0.5–1 mL hydrolysates and were both adjusted to the same OD_600_. Then various ratios of AY3 and BLF2 cells (0.5:1, 1:1, 1.5:1, 2:1, etc.) were inoculated into the DGS or algae hydrolysates at a final concentration of 20% (v/v). The induction and fermentation was performed as described above.

### Analytical methods

To determine the concentrations of glucose, xylose and arabinose in the medium, as well as the products such as isobutanol and ethanol, culture of the grown cells was centrifuged at 13,000 rpm for 10 min and 5 mL of the supernatant was injected into an Agilent HPLC system (1100 Series) equipped with the Rezex ROA-Organic Acid Sugar column (Phenomenex, CA). Other fusel alcohols, including 2-methyl-1-butanol, 3-methyl-1-butanol, 2-phenylethanol were extracted with ethyl acetate at the ratio of 1:1 (fermentation broth: ethyl acetate) with 2-methyl-1-pentanol as the internal reference. The ethyl acetate layer was collected for GC–MS analysis. One microliter of sample was injected into the injection port (250 °C) of an Agilent gas chromatography 6890N equipped with a 30 m × 0.25 mm DB-WAXetr capillary column with a film thickness of 0.5 µm. The temperature of the column was programmed as follows: 40 °C for 4 min, increasing to 65 °C at 10 °C/min and holding for 10 min, then increasing to 300 °C at 65 °C/min and holding for 5 min. The carrier gas was ultra-high purity helium at a constant flow rate of 1.5 mL/min. The chromatograph was coupled to a quadrupole MS 5975B. Spectral components were searched against the Wiley275 mass spectral library.

The total amino acids and proteins in the pretreated DGS and *Nannochloropsis* sp. hydrolysates before and after fermentation were determined using the ninhydrin assay [20]. The total carbohydrates in algae hydrolysates were determined by the phenol–sulfuric acid method [21] using a glucose standard.

### Real time quantitative PCR

Primers for the species-specific sequences of BLF2 and AY3 strains were designed for the quantitative PCR reaction. Primers 5′-GCTTTAATGAGTGGAATCGCC-3′ and 5′-GATGCAATGTTCTGGCTAACG-3′ were used to specifically amplify the *agaE* gene of *E. coli* BLF2 strain and primers 5′-GTGGAAAGAGGGCGATAAGAG-3′ and 5′-TCATGACGTTGGTAGAAGCG-3′ were used for the specific amplification of the *malB* gene of AY3 strain.

The q-PCR assays were carried out with the CFX96 Real-time PCR system with a C1000 Thermal Cycler (Bio-Rad, CA). The reaction mixture of 20 µL final volumes contained 1 µL DNA template, 0.15 µM each respective primer, and 10 µL of SYBR Green Master Mix (Bio-Rad, CA). All amplifications were carried out in optical grade 96 well plates (Fisher Scientific, MA) with an initial step at 98 °C for 3 min followed by 35 cycles of 98 °C for 15 s, 59 °C for 30 s. At the completion of each run, melting curves for the amplicons were measured by raising the temperature 0.5 °C from 65 to 95 °C while monitoring fluorescence. The specificity of the PCR amplification was checked by examining the melting curve for T_m_ and the lack of non-specific peaks. All tests were conducted in triplicate.

### Cell number determination in the co-culture

The cell numbers of *E. coli* BLF2 and AY3 in the co-culture were determined by the PCR-based multiple species cell counting method as described by Huang et al. [22]. To prepare the reference mixed samples, *E. coli* BLF2 and AY3 were grown overnight in 3 mL LB medium respectively. Then their individual colony forming units per mL (CFU/mL) were determined using serial dilutions and plating method. The genomic DNA of the individual samples was extracted using the Quick-DNA Fungal/Bacterial Miniprep Kit (Zymo Research, CA) and the same amount (by volume) of DNA solution extracted from the two species was mixed and the threshold cycle *C*_*T,R*_ was determined by quantitative PCR. For the unknown mixed samples, the genomic DNA of 2 mL fermentation culture was extracted and q-PCR was performed to determine the *C*_*T,X*_ as described above.

The cell numbers of *E. coli* BLF2 and AY3 in the co-culture samples during the fermentation process are determined by the following equation modified from [22] (the genomic DNA of the reference samples and unknown co-culture samples have the same dilution for q-PCR reaction):

$$N_{X} = \left( {1 + E} \right)^{{C_{T,R} - C_{T,X} }} \times CFU_{R} \times V_{R} ,$$

where *N*_*X*_ = cell number of *E. coli* BLF2 or AY3 in the co-culture; *E* = amplification efficiency of the q-PCR reaction using the primers specific to BLF2 or AY3; *C*_*T,R*_ = the threshold cycles (*C*_*T*_) of q-PCR for BLF2 or AY3 in the reference sample; *C*_*T,X*_ = the threshold cycles (*C*_*T*_) of q-PCR for BLF2 or AY3 in the unknown co-culture sample; *CFU*_*R*_ = the cell concentration of BLF2 or AY3 reference sample; *V*_*R*_ = the volume of processed reference cells for DNA extraction.

## Results

### Strain development

*Escherichia coli* strain AY3 previously developed in our lab [17] was used for the conversion of the protein fractions in the DGS hydrolysates into C4 and C5 fusel alcohols. *E. coli* AY3 is an improved strain of *E. coli* YH83 which was engineered to deaminate proteins and was able to utilize amino acids as the sole carbon source for growth [17, 19]. The mutant *E. coli* YH83 was the YH40 strain (BW25113/F′ [traD36, proAB^+^, lacI^q^ZΔM15] ΔglnA, ΔgdhA ΔluxS ΔlsrA) overexpressing isobutanol biosynthesis pathway genes (*alsS*-*ilvC*-*ilvD*-*kivd*-*yqhD*) and amino acids degradation genes (*ilvE, ilvA, sdaB, avtA* and *LeuDH*) in three separate plasmids pYX68, pYX90 and pYX97 (Table 1) [19]. The cofactor specificity of two key enzymes in the alcohol metabolic pathway has been modified through the directed evolution approach to create AY3 strain with improved fusel alcohol production yield [17].

**Table 1:** Bacterial strains and plasmids used in this study

Designation	Relevant characteristics	Source/references
Plasmids		
pYX68	pSC101 ori; Chl^R^; P*rrnB*; *ilvE*-*ilvA*-*sdaB*	[19]
pYX90	p15A ori; Spect^R^; *P*_L_lacO_1_; *alsS*-*ilvC*-*ilvD*-*avtA*	[19]
pYX97	ColE1 ori; Amp^R^; *P*_L_lacO_1_; *leuDH*-*kivd*-*yqhD*	[19]
pLF101	p15A ori; Spect^R^; *P*_L_lacO_1_; *alsS*-*ilvC*-*ilvD*	This work
pLF102	ColE1 ori; Amp^R^; *P*_L_lacO_1_; *kivd*-*yqhD*	This work
Strains		
*E. coli* DH5α	*lacZ*DM15 *recA*	NEB
*E. coli* YH40	BW25113/F′ [*traD*36, *proAB* ^+^, lacI^q^ ZΔM15] derivative with enhanced ability of amino acid utilization and with Δ*glnA*, Δ*gdhA*, Δ*lsrA*	[19]
*E. coli* AY3	*E. coli* YH40 with plasmids pYX68, pYX90 with the mutant genes and pYX97 with the mutant genes	[17, 19]
*E. coli* B	Prototroph	ATCC 11303
*E. coli* B01	*E. coli* B *Δldh*:: cam^+^	This work
*E. coli* BLF2	*E. coli* B01 with plasmids pLF101 and pLF102	This work

*Escherichia coli* strain B (ATCC 11303) was selected as the wild type in this study for constructing the fusel alcohol production strain for carbohydrate utilization, because of its natural ability to metabolize glucose as well as xylose sugars [23]. Therefore, this strain offers the opportunity to convert both hexose and pentose sugars present in the DGS hydrolysates. First, the gene encoding lactate dehydrogenase (*ldh*) was deleted from the chromosome of *E. coli* strain B using the technique of one-step disruption of chromosomal genes and was replaced with the chloramphenicol resistance gene (Cm^R^) from the plasmid pKD3 [18]. The resulting strain *E. coli* B01 had resistance to chloramphenicol, which enabled it to be co-cultured with the protein conversion strain *E. coli* AY3 that requires three antibiotic selectable markers (Cm^R^, Amp^R^, Sm^R^) to retain the plasmids. Two plasmids for introducing the pathway into *E. coli* B01 strain for isobutanol production from 2-keto acid precursors were constructed. Plasmid pLF101(Sm^R^) contained the genes encoding for acetolactate synthase (AlsS) from *Bacillus subtilis*, acetohydroxy acid isomeroreductase (IlvC) and dihydroxyacid dehydratase (IlvD) from *E. coli* [24] and the second plasmid pLF102 (Amp^R^) contained the genes encoding for 2-ketoacid decarboxylase (Kdc) from *Lactococcus lactis* and alcohol dehydrogenase (Adh) from *E. coli* [24]. These two plasmids were co-transformed into *E. coli* B01 strain and the resulting strain. *Escherichia coli* BLF2 (Table 1) overexpressed the five genes involved in the isobutanol production pathway. Therefore, pyruvate produced from glucose and xylose is converted by AlsS, IlvC and IlvD to 2-ketoisovalerate (KIV) which is further converted to isobutanol by Kdc and Adh (Additional file 1: Figure S1). Although Kdc from *L. lactis* has the highest specific activity towards 2-ketoisovalerate, it can also utilize several other 2-keto acids as substrates with lower specific activities [25]. Therefore, besides isobutanol, other fusel alcohols such as 2-methyl-1-butanol and 3-methyl-1-butanol may also be produced from other 2-keto acid precursors such as 2-ketoisocaproate (KIC) and 2-ketomethylvalerate (KMV) respectively by Kdc and Adh (Additional file 1: Figure S1).

### Fermentation of glucose and xylose sugars by *E. coli* BLF2

**Fig. 2:**
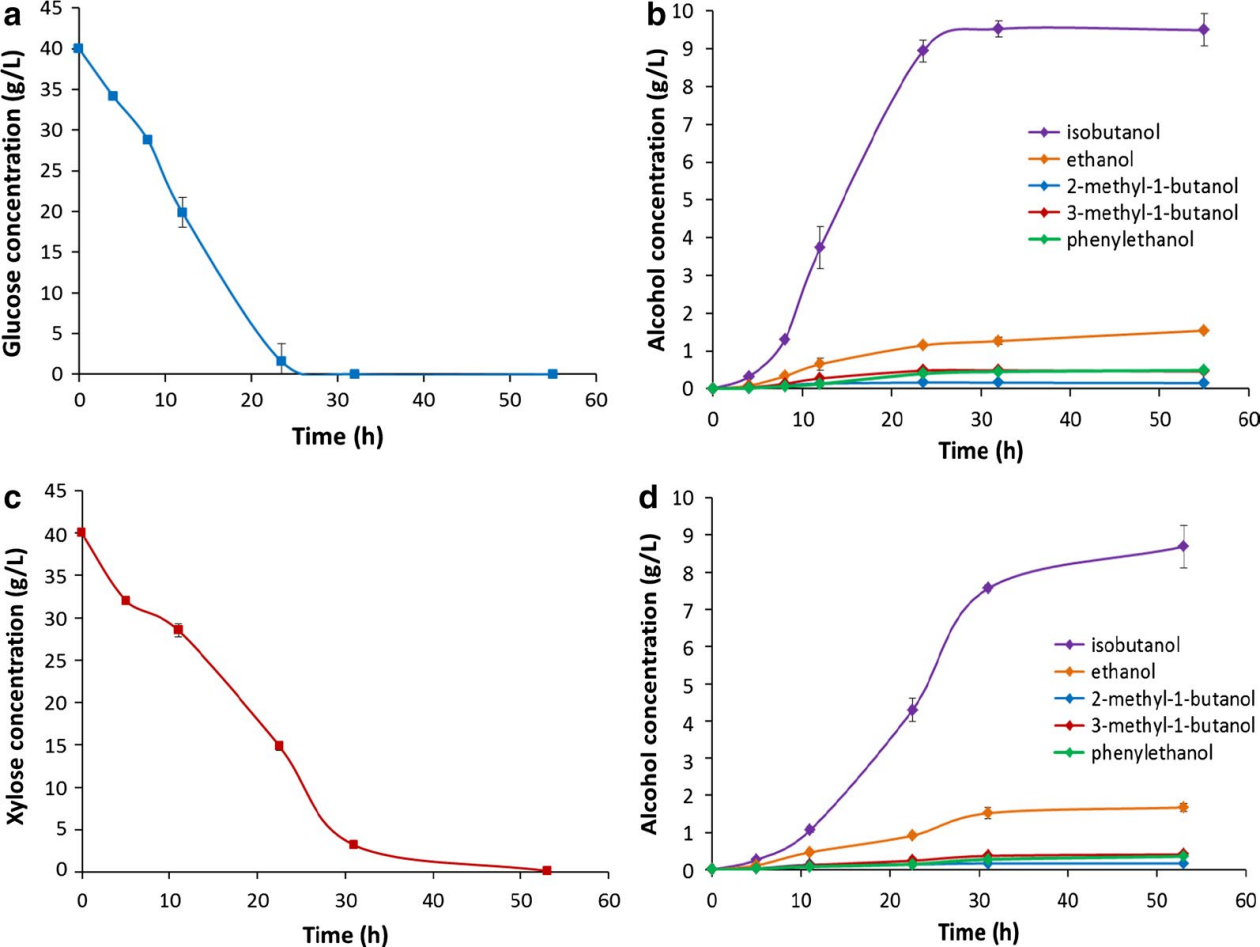
Fermentation of glucose or xylose as a sole carbon source by *E. coli* BLF2. **a** Time-dependent glucose concentration in the medium during fermentation. **b** Kinetic profile of fusel alcohol production from glucose. **c** Time-dependent xylose concentration in the medium during fermentation. **d** Kinetic profile of fusel alcohol production from xylose

To evaluate isobutanol production yield from the engineered carbohydrate conversion strain *E. coli* BLF2, we used synthetic media which contained either glucose or xylose or glucose and xylose mixture as the sole carbon source for the cell growth. As analyzed by HPLC and GC–MS, the majority of the fermentation product of *E. coli* BLF2 was isobutanol (Fig. 2b). Other alcohols such as 2-methyl-1-butanol, 3-methyl-1-butanol, 2-phenylethanol and ethanol were also observed. At the end of the shake flask fermentation, a total of 12.1 g/L mixed fusel alcohols were produced from initial 40 g/L glucose, including 9.5 g/L isobutanol which comprised 80% of the alcohol mixture (Fig. 2b). An average volumetric productivity of about 0.47 g/L h for the total alcohols was achieved when glucose was used as the sole carbon source. When growing in xylose medium, the xylose utilization rate was about 30% lower than glucose (Fig. 2a, c). Alcohol production with an average productivity of 0.32 g/L h was obtained which was similarly ~ 30% lower than that from glucose (Fig. 2b, d).

**Fig. 3:**
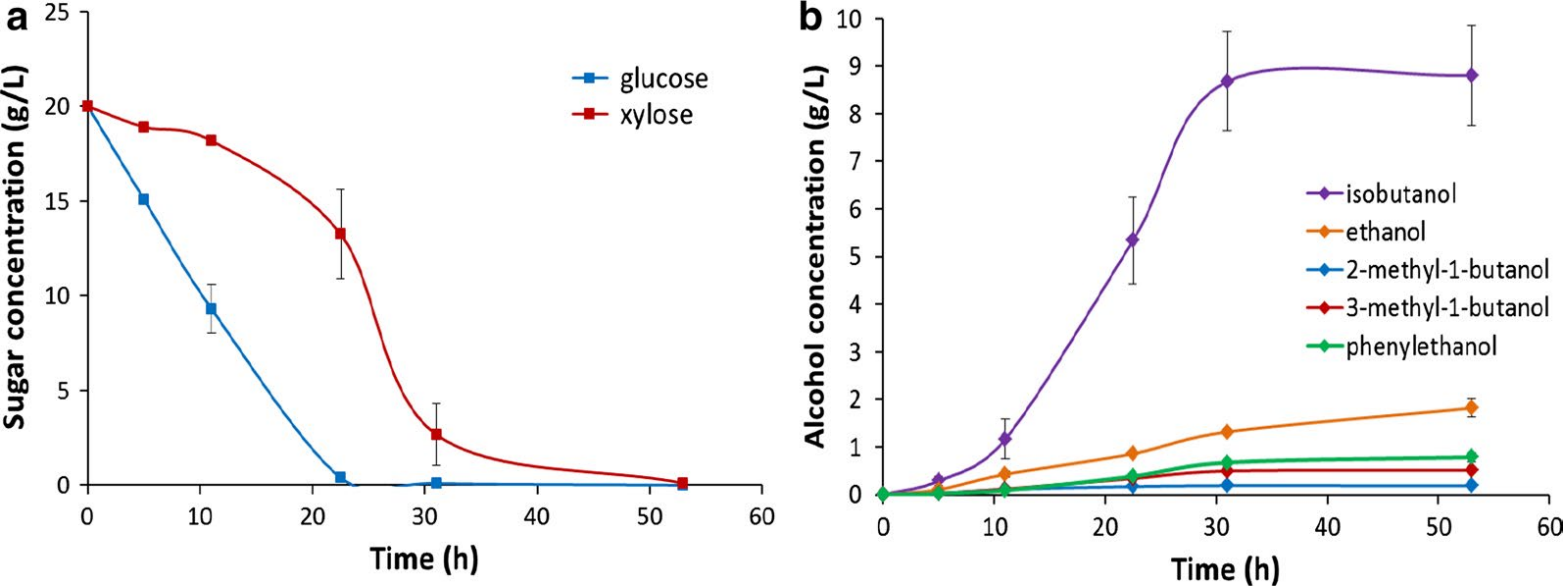
Fermentation of a glucose and xylose mixture by *E. coli* BLF2. **a** Time-dependent glucose and xylose concentrations during the mixed sugar fermentation. **b** Kinetic profile of fusel alcohol production during the fermentation

When sugar mixtures containing 20 g/L glucose and 20 g/L xylose was used as the growth medium, the cells preferably utilized glucose, and the utilization rate of xylose was slower than when it was fermented as a sole carbohydrate source (Fig. 3a), which suggests activation of carbon catabolite repression mechanisms [26]. Glucose was completely exhausted after 20 h of cultivation while xylose was completely consumed after 50 h (Fig. 3a). The volumetric productivity for the total fuel alcohols from the sugar mixture was about 0.37 g/L h (Fig. 3b) which was lower than that from glucose but higher than when xylose was used as a sole carbon source.

### DGS fermentation by *E. coli* BLF2

**Fig. 4:**
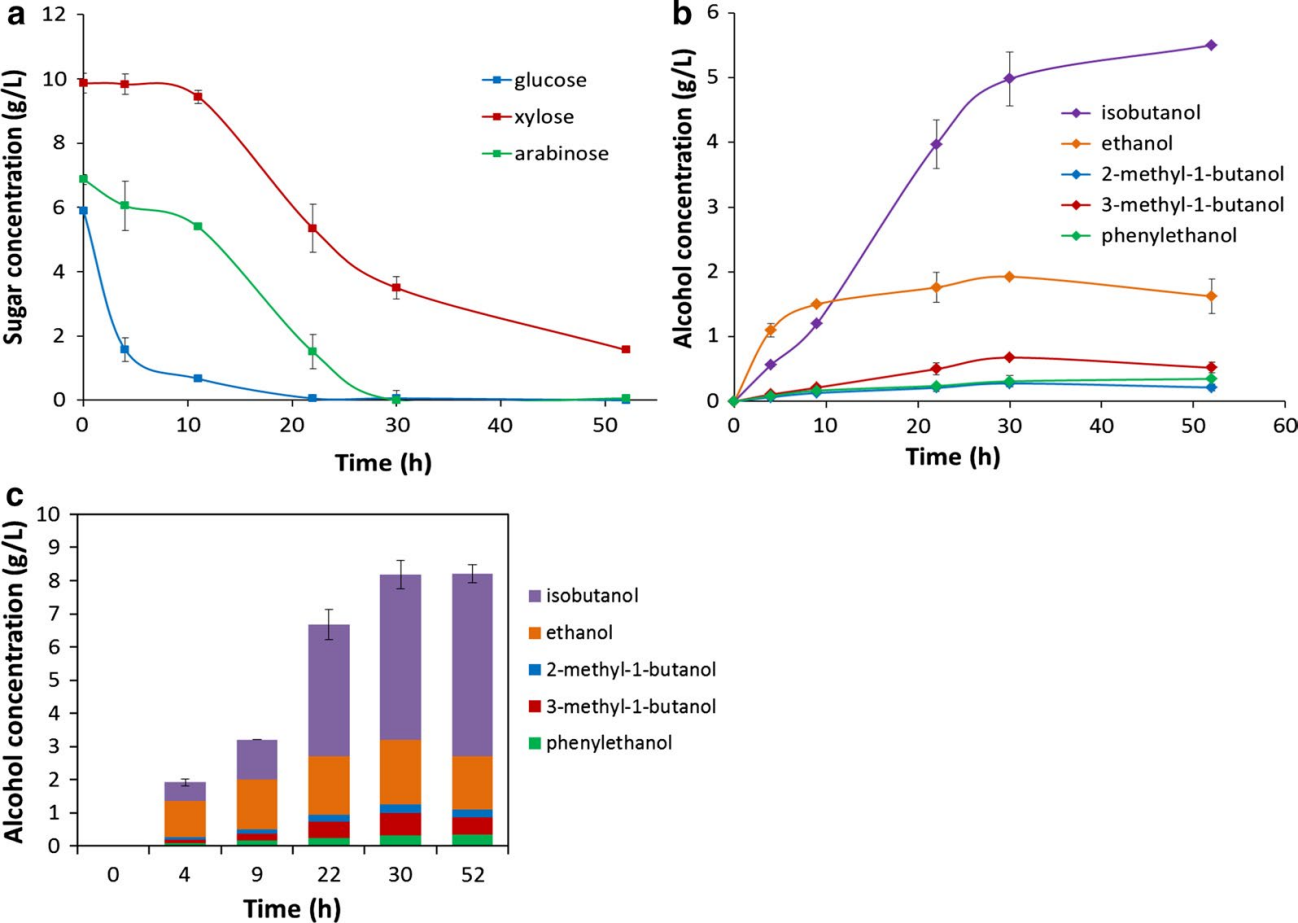
Fermentation of pretreated DGS hydrolysates by *E. coli* BLF2. **a** Time-dependent glucose, xylose and arabinose concentrations during the fermentation. **b** Kinetic profile of fusel alcohol production during the fermentation. **c** Bar graph showing the compositions of the mixed fusel alcohols produced during the fermentation

The kinetics of fusel alcohol production from carbohydrates present in DGS hydrolysates by *E. coli* BLF2 was evaluated from a time series study. The distillers’ grains samples obtained from a bioethanol company (Aemetis, Inc.) were pretreated with 4% sulfuric acid at 8.5% solids loading. Dilute-acid based methods have been used for pretreatment of a variety of lignocellulosic substrates for facilitating conversion of oligosaccharides to monomeric sugars suitable for bacterial fermentation [27–29]. As analyzed by HPLC, the DGS hydrolysates after dilute-acid pretreatment contained 6 g/L glucose, 10 g/L xylose and 7 g/L arabinose. The pretreated DGS hydrolysates without any additional supplement were used directly for BLF2 fermentation. During the fermentation course, glucose was preferentially utilized by the cells, and the uptake of xylose and arabinose was inhibited until glucose concentration was significantly attenuated (Fig. 4a), which suggests the inhibition of xylose and arabinose metabolism in the presence of glucose (i.e. catabolite repression). At the end of the 52-h fermentation, glucose and arabinose was completely consumed while 84% of the total xylose in the hydrolysates was utilized with about 1.6 g/L unutilized. The conversion of the sugar fraction in the DGS hydrolysates by *E. coli* BLF2 resulted in a total of 8.2 g/L fusel alcohols including 5.5 g/L isobutanol which was 67% of the mixed alcohols (Fig. 4b, c).

**Fig. 5:**
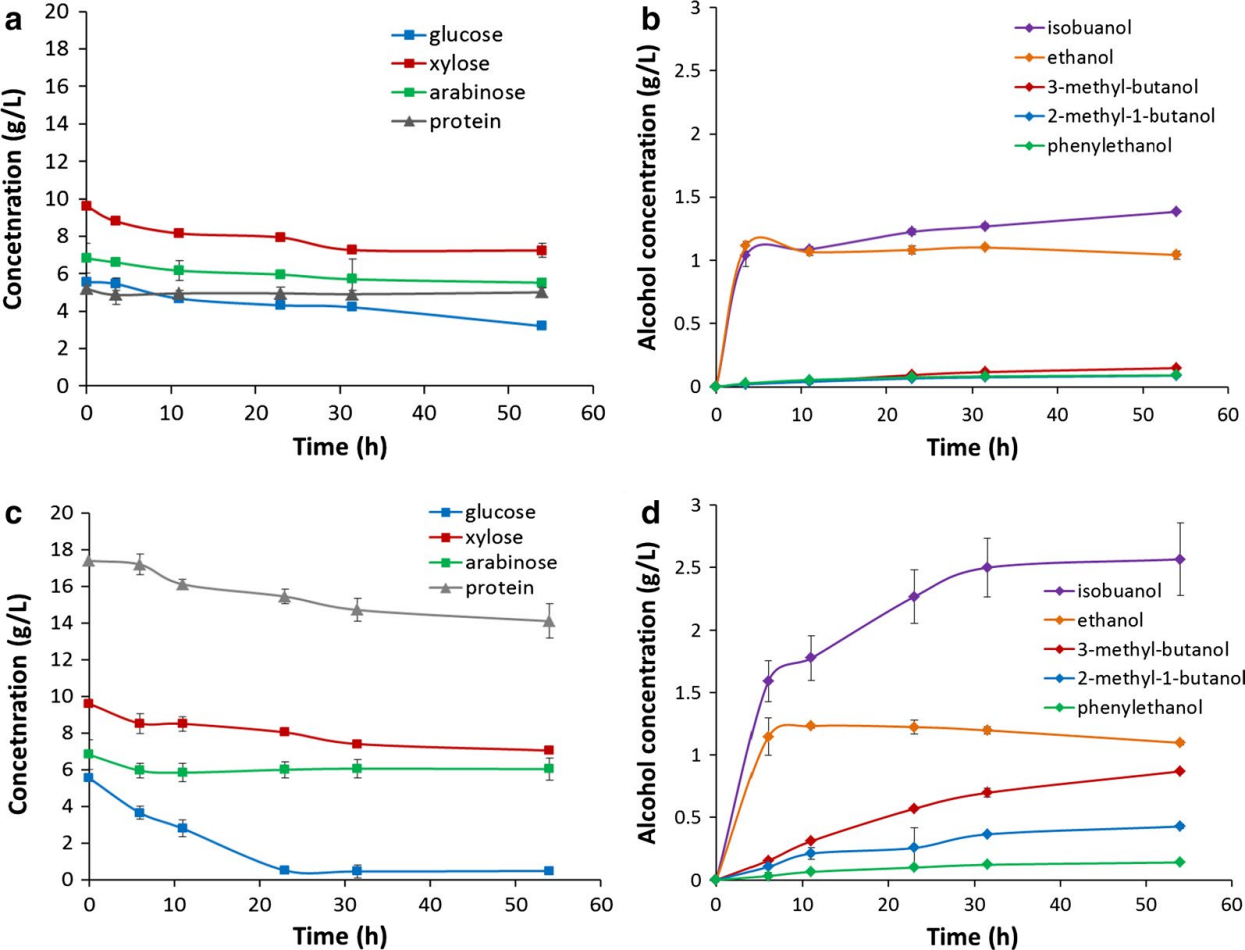
Fermentation of pretreated DGS hydrolysates with and without Pronase digestion by *E. coli* AY3 strain. **a** Time-dependent sugar and protein concentrations during the fermentation of undigested DGS hydrolysate. **b** Kinetic profile of fusel alcohol production during the fermentation of undigested DGS hydrolysate. **c** Time-dependent sugar and protein concentrations during the fermentation of DGS hydrolysate with Pronase digestion. **d** Kinetic profile of fusel alcohol production during the fermentation of DGS hydrolysate with protease digestion

The protein conversion strain *E. coli* AY3 was previously engineered to utilize amino acids as carbon source for growth [17, 19]. Although it could also use glucose for growth, AY3 strain showed very limited ability in utilizing pentose sugars (xylose and arabinose) in the DGS hydrolysates (Fig. 5a, c). In the undigested DGS hydrolysates, there was about 5 g/L amino acids which remained unconverted as determined by the ninhydrin method [20]. AY3 only consumed 42% glucose and produced 2.8 g/L mixed fusel alcohols from the DGS hydrolysates without digestion (Fig. 5a, b). After Pronase treatment, a total of 17.4 g/L free amino acid was released from the proteins in the DGS hydrolysates which can be utilized by *E. coli* AY3 as carbon source for growth. AY3 performed significantly better in the digested DGS hydrolysates and converted 3.3 g/L amino acids and 5.1 g/L glucose and produced a total of 5.1 g/L mixed fusel alcohols (Fig. 5c, d).

### ‘One-pot’ bioconversion of DGS hydrolysate by *E. coli*–*E. coli* co-cultures

Based on the techno-economic impact of reducing unit operations and increasing net conversion yields of the whole biomass hydrolysate, we investigated the feasibility of simultaneous bioconversion of protein and carbohydrate fractions in a ‘one-pot’ fermentation by co-culturing the two strains *E. coli* BLF2 and AY3. In the co-culture, *E. coli* BLF2 was dedicated for conversion of hexose and pentose sugars in DGS hydrolysates into C4 and C5 fusel alcohols and *E. coli* AY3 was designated to convert DGS proteins into C4 and C5 fusel alcohols (Fig. 1).

**Fig. 6:**
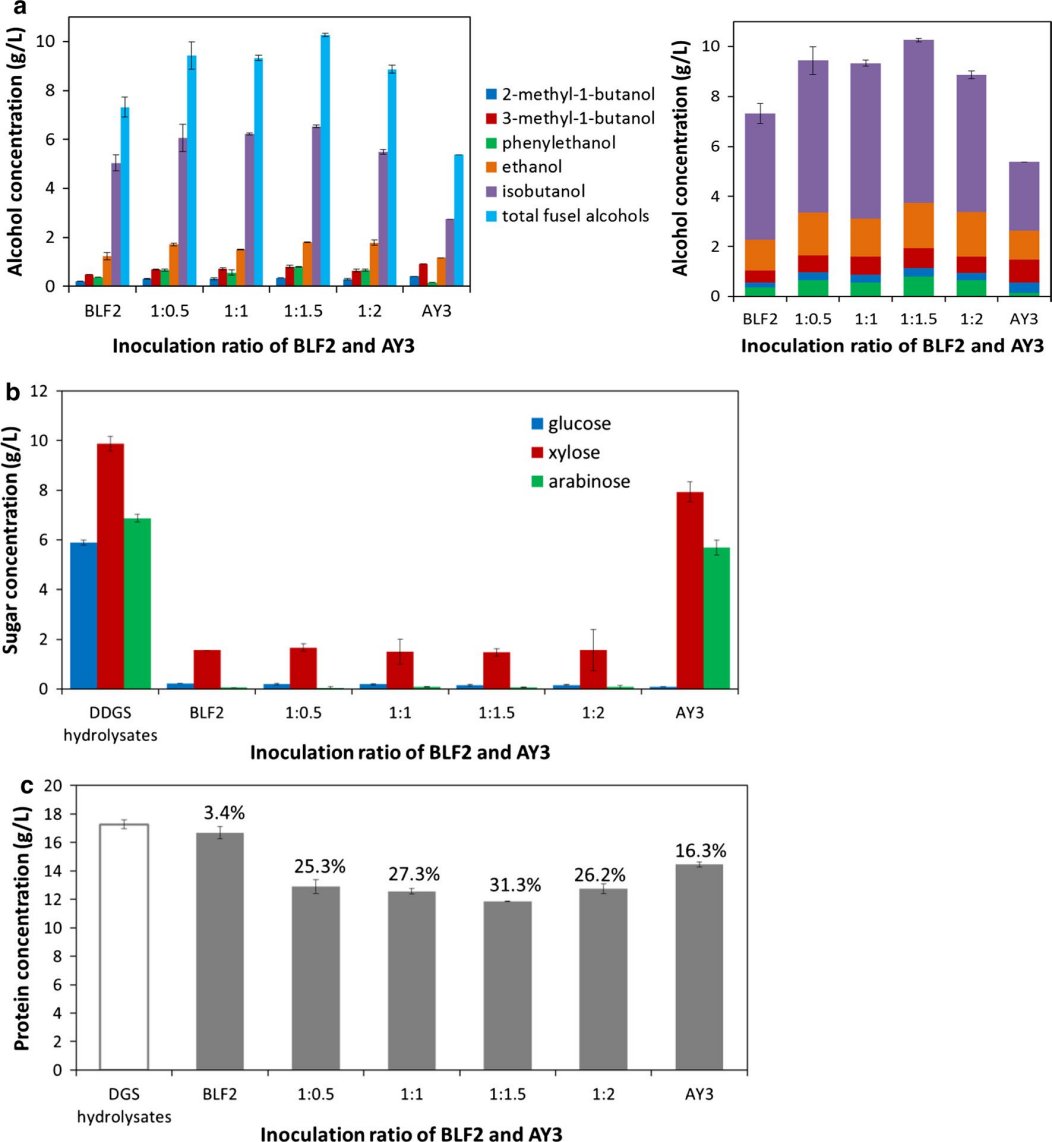
Conversion of DGS hydrolysates by the co-culture of *E. coli* BLF2 and AY3 at different inoculation ratios. **a** Fusel alcohol production at 52 h and its composition analysis. **b** The concentration of sugars in the hydrolysates before and after fermentation. **c** The concentration of proteins in the hydrolysates before and after fermentation. Numbers provided above the bars indicate the percentages of protein converted

After dilute-acid pretreatment, the DGS hydrolysates were digested with Pronase to hydrolyze the proteins to monomeric amino acids or short peptides that can be readily utilized for co-culture fermentation. To optimize the inoculation ratio between the two strains in the co-culture system, the fusel alcohol yields were investigated under different initial BLF2/AY3 inoculation ratios at 0.5:1, 1:1, 1.5:1 and 2:1 as well as that single strains of BLF2 or AY3 alone. As shown in Fig. 6a, when co-culture of the two strains were grown on DGS hydrolysates at an inoculation ratio of 1:1.5, the highest titer of total fusel alcohols up to 10.3 g/L was produced, including 6.5 g/L isobutanol which comprised 63.1% of the total alcohols. Correspondingly, the co-culture with the inoculation ratio of 1:1.5 consumed the highest total amount of carbohydrates and proteins in the hydrolysates (Fig. 6b, c). The co-culture system resulted in nearly complete consumption of the glucose and arabinose and consumption of 85.1% of the xylose in the DGS hydrolysates (Fig. 6b). 31.3% of the total proteins in the hydrolysates were also converted by the co-culture with the inoculation ratio of 1:1.5 (Fig. 6c). The co-cultures involving the two *E. coli* strains with different inoculation ratios all produced higher quantities of fusel alcohols than the monoculture BLF2 and AY3 alone, which indicated that both of the strains were contributing to the substrate conversion and fusel alcohol production. Although *E. coli* AY3 could uptake amino acids as the sole carbon source for growth, AY3 also utilized glucose for growth when monomeric sugars were present (Fig. 6b). Only 16.3% of the protein fraction in the DGS hydrolysates was converted by AY3 monoculture when sugars and proteins were both present in the hydrolysates (Fig. 6c). In contrast, higher conversion rates of proteins were achieved by the co-cultures, which indicated that the competition of BLF2 strain for sugar as carbon source induced AY3 to utilize more proteins for growth and alcohol production.

### Bioconversion of the algae hydrolysates by *E. coli* co-cultures

**Fig. 7:**
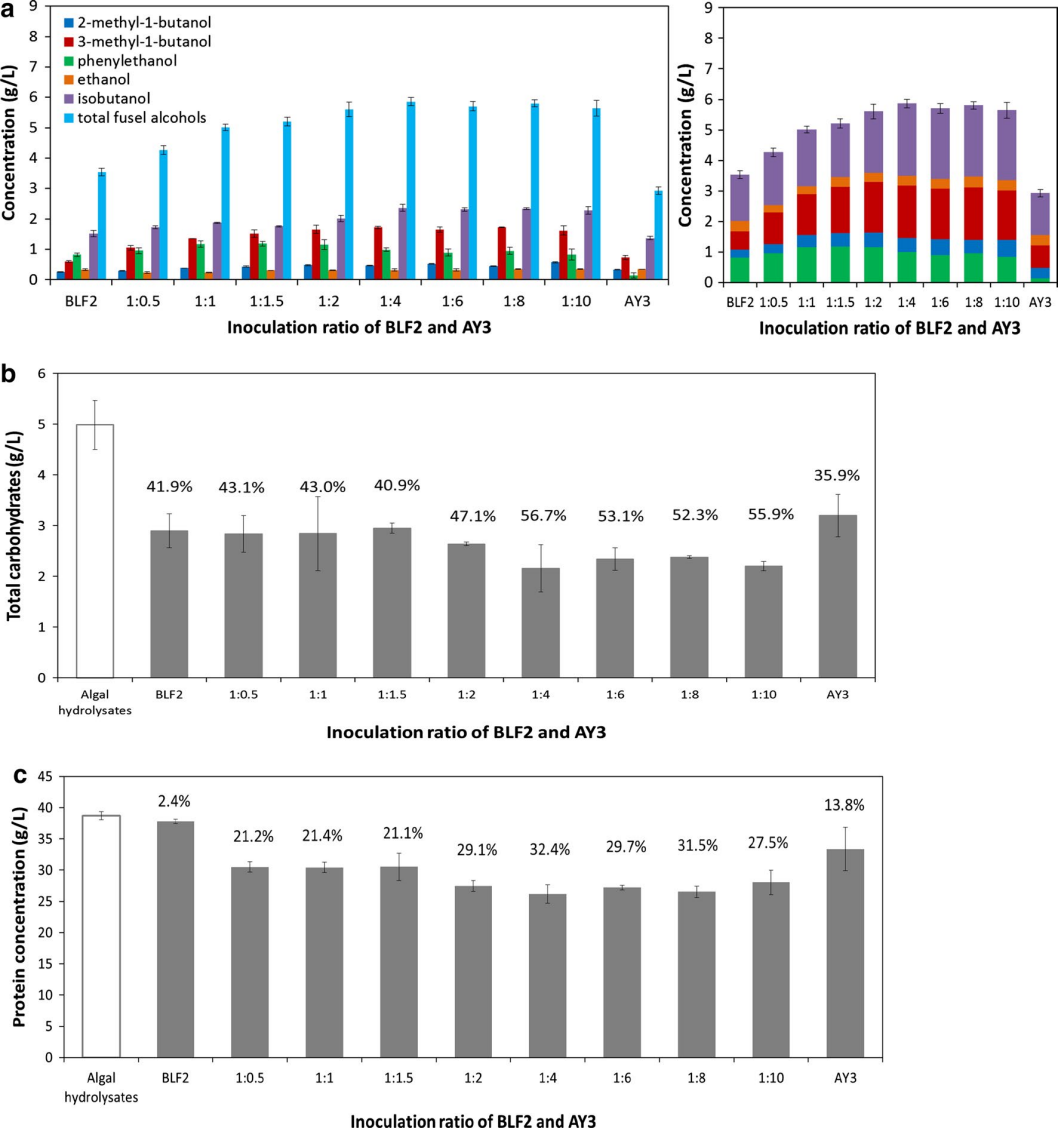
Conversion of *Nannochloropsis* sp. algae hydrolysates by the co-culture of *E. coli* BLF2 and AY3 at different inoculation ratios. **a** Fusel alcohol production at 48 h and its composition analysis. **b** The concentration of sugars in the hydrolysates before and after fermentation. The numbers showed the percentages of carbohydrate converted. **c** The concentration of proteins in the hydrolysates before and after fermentation. Numbers provided above the bars indicate the percentages of protein converted

We further investigated the applicability of this microbial co-culture for the bioconversion of alternative hydrolysates that are rich in carbohydrates and proteins, a prominent example of which is microalgae. *Nannochloropsis* sp. hydrolysates produced from dilute acid and enzymatic pretreatment were inoculated with the BLF2-AY3 co-cultures at variable inoculation ratios. The algae hydrolysates were different from the DGS hydrolysates in that the latter contained a total of ~ 23 g/L fermentable sugars and ~ 17 g/L proteins, whereas the algae hydrolysates had a much higher fraction of proteins (~ 38 g/L) but much smaller amount of sugar with a total carbohydrate of ~ 5 g/L. As shown in Fig. 7a, the co-culture with an inoculation ratio of 1:4, 1:6, 1:8 and up to 1:10 of BLF2 and AY3 produced higher amount of fusel alcohols and the 1:4 ratio led to the highest amount of mixed fusel alcohols, 5.9 g/L. The composition of the fusel alcohols products from algae hydrolysates included isobutanol (40.3% (w/w)) and mixed isopentanols (2-methyl-1-butanol and 3-methyl-1-butanol (37.3% (w/w)), indicating significant enrichment of the C5 alcohols compared to the product spectrum produced from DGS, where isobutanol was the major product (63.1% (w/w)). Since the alcohol mixture produced by AY3 monoculture had a higher fraction of isopentanol than that produced by BLF2 monoculture (Figs. 6a, 7a), this compositional change of the fuel products suggested that AY3 may have played a more significant role in the conversion of the algae hydrolysates in the co-culture than in DGS hydrolysates, which was also in agreement with the fact that algae hydrolysates had more proteins available for AY3 to utilize than in DGS hydrolysates. Up to 32.4% of the initial 38.7 g/L proteins in the algae hydrolysates were converted by the co-culture with an inoculation ratio of 1:4 (Fig. 7c).

### Dynamics of the co-culture by q-PCR analysis

To differentiate BLF2 and AY3 strain in the co-culture and to monitor the cell number of each species during fermentation, specific primers targeting the unique genes in the chromosome of BLF2 and AY3 strain were designed. Although the *E. coli* strains have high nucleotide sequence homology and similar genome organization, BLF2 was engineered from wild-type B strain while AY3 was derived from the K-12 strain. *E. coli* B strain is deficient for *malB* gene encoding for the maltose high affinity receptor which is present in the K-12 strain [30], while K-12 strain lacks the IID domain of the *N*-acetyl-galactosamine transporter (*agaE*) [31]. Therefore, the primers specific for *malB* and *agaE* were used to specifically target AY3 and BLF2 respectively. The specificity of the primers and validation of the q-PCR test was confirmed (Additional file 1: Figure S2). The parameters needed for calculating the cell numbers of BLF2 and AY3 in the co-culture as described in “Methods” section were also determined (Additional file 1: Figure S3, Table S1).

The cell numbers of BLF2 and AY3 in the co-culture at the end of fermentation were determined by the newly developed q-PCR based quantification method. As Tables 2 and 3 show, the cell number of AY3 grown in the DGS and algae hydrolysates was 3-tenfold lower than that of BLF2 alone, which indicates that AY3 grew more slowly than BLF2 strain. In the co-culture mixture, as the initial inoculation ratio of BLF2/AY3 decreased, the final BLF2/AY3 ratio in the co-culture at the end of fermentation also decreased in both of the hydrolysates. When more cells of BLF2 than AY3 were inoculated, for example at the 1:0.5 inoculation ratio, the final BLF2/AY3 ratio of 43.9 and 59.3 was observed for the DGS and algae hydrolysates, respectively (Tables 2, 3). Only when more AY3 was initially inoculated, the difference of the cell numbers of the two species at the end of the fermentation was significantly reduced. When BLF2 and AY3 were inoculated at the ratio of 1:1.5 and 1:2, the final ratio of BLF2/AY3 reduced to 1.5 and 1.2, respectively (Table 2). Similarly, the difference of the cell numbers between the two species was reduced to less than fourfold at 48-h fermentation in the algae hydrolysates when AY3 was inoculated at least four times more cells than BLF2 in the co-culture (Table 3). The fusel alcohols produced by the co-culture at these inoculation ratios were higher than others, which suggests that a balanced population of the two strains during fermentation is important for the engineered co-culture to achieve higher fusel titers.

**Table 2:** Individual populations of BLF2 and AY3 in the co-culture at the end of fermentation of DGS hydrolysates based on q-PCR analysis

Initial BLF2/AY3 inoculation ratio	Average cell number (cell/mL) in the co-culture at 52 h		Final BLF2/AY3 ratio in the co-culture at 52 h
	BLF2	AY3	
BLF2 alone	3.2 × 10^10^	–	–
1:0.5	1.8 × 10^10^	4.1 × 10^8^	43.9
1:1	2.7 × 10^10^	6.9 × 10^8^	39.1
1:1.5	8.2 × 10^9^	5.6 × 10^9^	1.5
1:2	1.9 × 10^9^	1.6 × 10^9^	1.2
AY3 alone	–	3.1 × 10^9^	–

**Table 3:** Individual populations of BLF2 and AY3 in the co-culture at the end of fermentation of algae hydrolysates based on q-PCR analysis

Initial BLF2/AY3 inoculation ratio	Average cell number (cell/mL) in the co-culture at 48 h		Final BLF2/AY3 ratio in the co-culture at 48 h
	BLF2	AY3	
BLF2 alone	2.0 × 10^9^	–	–
1:0.5	3.5 × 10^9^	5.9 × 10^7^	59.3
1:1	2.8 × 10^9^	8.8 × 10^7^	31.8
1:1.5	1.9 × 10^9^	9.3 × 10^7^	20.4
1:2	1.5 × 10^9^	2.5 × 10^8^	6.0
1:4	1.5 × 10^9^	4.0 × 10^8^	3.8
1:6	9.6 × 10^8^	2.8 × 10^8^	3.4
1:8	1.5 × 10^9^	4.6 × 10^8^	3.3
1:10	1.2 × 10^9^	6.5 × 10^8^	1.8
AY3 alone	–	7.4 × 10^8^	–

**Fig. 8:**
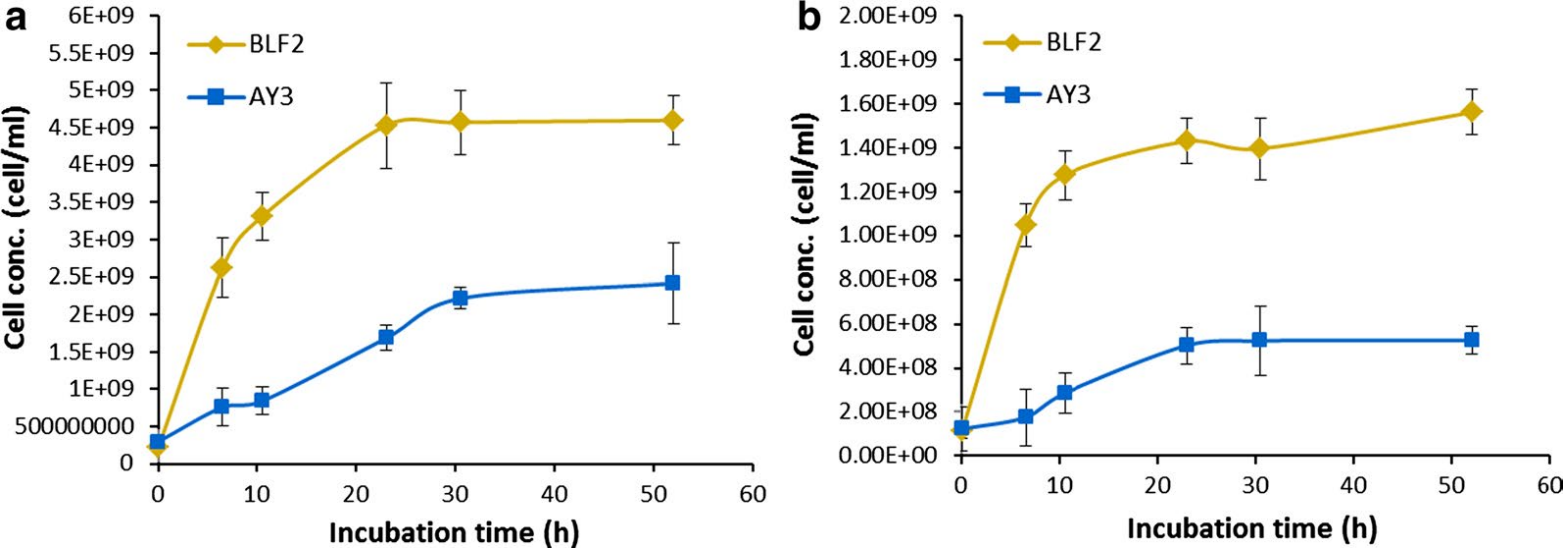
Growth dynamics of individual populations in the co-culture during the fermentation of hydrolysates analyzed by the q-PCR quantification method. **a** DGS hydrolysate with a BLF2/AY3 inoculation ratio of 1:1.5. **b** Algae hydrolysate with a BLF2/AY3 inoculation ratio of 1:4

The q-PCR quantification method also provided the temporal profile of cell growth for the two *E. coli* strains in the co-culture during fermentation. Samples of different time points during fermentation of the DGS hydrolysates with the initial BLF2/AY3 inoculation ratio of 1:1.5 and the algae hydrolysates with the inoculation ratio of 1:4 were collected respectively and the cell numbers were determined (Fig. 8). In both of the hydrolysates, the cell number of the two strains continuously increased until reaching plateau, which indicated that despite the growth rate difference between the two strains, the co-culturing didn’t adversely affect the growth of each strain. Although BLF2 appeared as the dominant species in the co-culture, AY3 strain was not eliminated during the fermentation. In fact, the final cell numbers of AY3 in the co-cultures at proper inoculation ratios of BLF2/AY3 were no less than the cell number of AY3 monoculture in the hydrolysates (Tables 2, 3).

## Discussion

DGS, the major coproduct from the bioethanol industry, is produced in large and increasing quantities annually. Efficient valorization of DGS to support starch bioethanol process viability requires processes to convert both of the major DGS biochemical pools—proteins and carbohydrates—to value-added products. In this study, we developed a microbial factory to convert both the protein and carbohydrate fractions of DGS to advanced biofuels. These results should support improvement of the techno-economic feasibility and net energy return of the first-generation bioethanol process since up to ~ 30% more fuel products can be produced from the same amount of corn. This integrated carbohydrate and protein conversion platform is versatile for the bioconversion of other carbohydrate and protein rich biomass, which was demonstrated using microalgae biomass. The mixed fusel alcohols that were produced contained primarily isobutanol and other higher carbon numbers alcohols, including 2-methyl-1-butanol, 3-methyl-1-butanol and 2-phenylethanol. It was previously shown that mixed alcohol forms (especially C_3_–C_5_) provide increased energy densities and other improved physical properties (e.g. reduced water solubility and corrosivity) than ethanol which can provide increased combustion efficiencies, reduced emission profiles, and improved compatibility with the existing liquid fuels infrastructure [32]. Therefore, mixed fusel alcohols have promising potential applications as a fuel blendstock in gasoline, diesel, jet fuel, heating oil or as a neat fuel of itself.

The microbial co-culture developed here, specifically the carbohydrate conversion strain and the protein conversion strain, allows the microbes to utilize multiple substrates and accomplish complex biosynthesis that is difficult to achieve by a single cell. Also it allows division of labor and reduction of the metabolic burden on each cell type. The isobutanol produced from glucose by the carbohydrate conversion strain *E. coli* BLF2 is higher than that which has been reported from a previous other study where the *E. coli* production strain included deletion of six genes involved in byproducts formation [24], suggesting superior capacity of *E. coli* strain B as a host for isobutanol production. We envision that the isobutanol yield from *E. coli* BLF2 strain can be further increased by optimizing the process conditions and strain engineering, e.g. deleting the competing pathways for the byproducts and removing any bottlenecks from the pathway.

In terms of protein conversion, up to 30% of proteins from both the DGS and algae hydrolysates were converted by the co-culture. The incomplete protein conversion in both hydrolysates is probably due to several facts. Firstly, the pretreated hydrolysates were directly used as the fermentation broth which may lack of some of the trace nutrients as in synthetic medium such as LB broth/; Additionally, potential fermentation inhibitors such as weak acids and furan derivatives [33] present in the hydrolysates may have inhibitory effect on the *E. coli* strain. Secondly, the protein conversion strain AY3 can only utilize 13 individual amino acids as the sole carbon source [19] which leads to the incomplete consumption of the proteins in the hydrolysates. Moreover, the carbohydrates present in the hydrolysates were also utilized as the carbon source for growth by AY3, which may reduce the consumption rate of proteins in the hydrolysates by the protein conversion strain AY3 compared with when only protein was available as the sole carbon source. This could be improved by using different inoculation strategies, i.e., inoculating AY3 following BLF2 in the co-culture when sugars in the hydrolysates are mostly consumed by the carbohydrate conversion strain BLF2 during fermentation. However, the fusel alcohol amino acid preference favors high abundance, low value amino acids such as glutamate and alanine [11]; therefore, isolation of the unutilized high value amino acids such as lysine, methionine and phenylalanine may provide a path toward a secondary high value co-product stream to further facilitate the process economics.

The q-PCR based cell quantification method developed here is a versatile tool for rapidly tracking the individual population in the mixed culture during fermentation. The protein conversion strain AY3 has much lower growth rate than the carbohydrate conversion strain BLF2, which is probably due to the fact that AY3 has more genetic modifications than BLF2, including deletion of several genomic genes [17, 19]. Previous studies have showed that multiple genomic deletions could cause decreases in the cell growth rate of *E. coli* [34]. When co-culturing two strains whose growth rates differ substantially, it’s likely that one species become the dominant population and therefore the population ratios often have to be optimized to obtain a stable culture so that one cell type does not eliminate the other [35]. In the case of our co-culture, although BLF2 cells did grow faster than AY3, the co-culturing of the two species didn’t eliminate the growth of AY3 (Fig. 8). This is probably due to the fact that BLF2 and AY3 don’t compete for pentose sugar and proteins as carbon source for growth, although they can both utilize glucose. Therefore, their substrate specificity allows the two strains to establish a stable co-culture system. Furthermore, the co-cultures at certain initial BLF2/AY3 inoculation ratios produced higher amount of fusel alcohols from the hydrolysates than others (Figs. 6a, 7a); q-PCR analysis clearly indicated that the difference between the cell numbers of BLF2 and AY3 in these co-cultures was minimized. The population dynamics analysis of the co-culture in this study demonstrated that changing the initial inoculation ratio is a simple and effective way to tune the co-culture population and that an optimized co-culture population is vital to achieve higher production yield by the engineered *E. coli* consortium.

## Conclusions

We demonstrated ‘one-pot’ bioconversion of the DGS hydrolysate into fusel alcohols using a microbial co-culture strategy incorporating two engineered *E. coli* strains. The carbohydrate conversion strain *E. coli* BLF2 was constructed from the wild type strain B and showed improved capability to produce fusel alcohols from hexose and pentose sugars compared to previous efforts. The co-culture with an inoculation ratio of 1:1.5 of *E. coli* BLF2 and AY3 achieved the highest total fuel titer of up to 10.3 g/L from DGS hydrolysates. Moreover, the integrated carbohydrate and protein conversion platform is also applicable for the bioconversion of other multi-substrate biomass such as algae hydrolysates. The detailed population dynamics study suggested that an optimized co-culture population ratio lead to more efficient ‘one-pot’ bioconversion of multiple substrates in the hydrolysates.

## Additional file

**Additional file 1.** Additional figures and table.

## References

[1] Renewable Fuels Association. World fuel ethanol production. 2016. http://www.ethanolrfa.org/resources/industry/statistics/#1454098996479-8715d404-e546. Accessed 9 Mar 2017.

[2] Bothast RJ, Schlicher MA (2005). Biotechnological processes for conversion of corn into ethanol. Appl Microbiol Biotechnol.

[3] Renewable Fuels Association. Industry resources: co-products. 2015. http://old.ethanolrfa.org/pages/industry-resources-coproducts. Accessed 9 Mar 2017.

[4] Renewable Fuels Association. Co-products. 2017. http://ethanolrfa.org/resources/industry/co-products. Accessed 9 Mar 2017.

[5] Liu K (2011). Chemical composition of distillers grains, a review. J Agric Food Chem.

[6] Zhou YJ, Buijs NA, Zhu Z, Qin J, Siewers V, Nielsen J (2016). Production of fatty acid-derived oleochemicals and biofuels by synthetic yeast cell factories. Nat Commun.

[7] Liao JC, Mi L, Pontrelli S, Luo S (2016). Fuelling the future: microbial engineering for the production of sustainable biofuels. Nat Rev Microbiol.

[8] Chubukov V, Mukhopadhyay A, Petzold C, Keasling J (2016). Synthetic and systems biology for microbial production of commodity chemicals : from target selection to scale-up. npj Syst Biol Appl.

[9] Liu F, Banta S, Chen W (2013). Functional assembly of a multi-enzyme methanol oxidation cascade on a surface-displayed trifunctional scaffold for enhanced NADH production. Chem Commun.

[10] Park M, Sun Q, Liu F, DeLisa MP, Chen W (2014). Positional assembly of enzymes on bacterial outer membrane vesicles for cascade reactions. PLoS ONE.

[11] Dueber JE, Wu GC, Malmirchegini GR, Moon TS, Petzold CJ, Ullal AV, Prather KLJ, Keasling JD (2009). Synthetic protein scaffolds provide modular control over metabolic flux. Nat Biotechnol.

[12] Bizukojc M, Dietz D, Sun J, Zeng AP (2010). Metabolic modelling of syntrophic-like growth of a 1,3-propanediol producer, *Clostridium butyricum*, and a methanogenic archeon, *Methanosarcina mazei*, under anaerobic conditions. Bioprocess Biosyst Eng.

[13] Qu Y, Feng Y, Wang X, Logan BE (2012). Use of a coculture to enable current production by *Geobacter sulfurreducens*. Appl Environ Microbiol.

[14] Zhang H, Pereira B, Li Z, Stephanopoulos G (2015). Engineering *Escherichia coli* coculture systems for the production of biochemical products. Proc Natl Acad Sci USA.

[15] Kim Y, Hendrickson R, Mosier NS, Ladisch MR, Bals B, Balan V, Dale BE (2008). Enzyme hydrolysis and ethanol fermentation of liquid hot water and AFEX pretreated distillers’ grains at high-solids loadings. Bioresour Technol.

[16] Sarathy SM, Oβwald P, Hansen N, Kohse-Höinghaus K (2014). Alcohol combustion chemistry. Prog Energy Combust Sci.

[17] Wu W, Tran-Gyamfi MB, Jaryenneh JD, Davis RW (2016). Cofactor engineering of ketol-acid reductoisomerase (IlvC) and alcohol dehydrogenase (YqhD) improves the fusel alcohol yield in algal protein anaerobic fermentation. Algal Res.

[18] Datsenko KA, Wanner BL (2000). One-step inactivation of chromosomal genes in *Escherichia coli* K-12 using PCR products. Proc Natl Acad Sci USA.

[19] Huo Y-X, Cho KM, Rivera JGL, Monte E, Shen CR, Yan Y, Liao JC (2011). Conversion of proteins into biofuels by engineering nitrogen flux. Nat Biotechnol.

[20] Friedman M (2004). Applications of the ninhydrin reaction for analysis of amino acids, peptides, and proteins to agricultural and biomedical sciences. J Agric Food Chem.

[21] Masuko T, Minami A, Iwasaki N, Majima T, Nishimura SI, Lee YC (2005). Carbohydrate analysis by a phenol–sulfuric acid method in microplate format. Anal Biochem.

[22] Huang R, Zhang J, Yang XF, Gregory RL (2015). PCR-based multiple species cell counting for in vitro mixed culture. PLoS ONE.

[23] Alterthum F, Ingram L (1989). Efficient ethanol production from glucose, lactose, and xylose by recombinant *Escherichia coli*. Appl Environ Microbiol.

[24] Atsumi S, Wu TY, Eckl EM, Hawkins SD, Buelter T, Liao JC (2010). Engineering the isobutanol biosynthetic pathway in *Escherichia coli* by comparison of three aldehyde reductase/alcohol dehydrogenase genes. Appl Microbiol Biotechnol.

[25] De La Plaza M, Fernández De Palencia P, Peláez C, Requena T (2004). Biochemical and molecular characterization of α-ketoisovalerate decarboxylase, an enzyme involved in the formation of aldehydes from amino acids by *Lactococcus lactis*. FEMS Microbiol Lett.

[26] Kim JH, Block DE, Mills DA (2010). Simultaneous consumption of pentose and hexose sugars: an optimal microbial phenotype for efficient fermentation of lignocellulosic biomass. Appl Microbiol Biotechnol.

[27] Noureddini H, Byun J (2010). Dilute-acid pretreatment of distillers’ grains and corn fiber. Bioresour Technol.

[28] Um B-H, Karim M, Henk L (2003). Effect of sulfuric and phosphoric acid pretreatments on enzymatic hydrolysis of corn stover. Appl Biochem Biotechnol.

[29] Zhu Y, Lee YYER (2004). Dilute-acid pretreatment of corn stover using a high-solids percolation reactor. Appl Biochem Biotechnol.

[30] Studier FW, Daegelen P, Lenski RE, Maslov S, Kim JF (2009). Understanding the differences between genome sequences of *Escherichia coli* B strains REL606 and BL21(DE3) and comparison of the *E. coli* B and K-12 genomes. J Mol Biol.

[31] Brinkkötter A, Klöß H, Alpert CA, Lengeler JW (2000). Pathways for the utilization of N-acetyl-galactosamine and galactosamine in *Escherichia coli*. Mol Microbiol.

[32] Jimeson RM, Radosevich MC, Stevens RR. Mixed alcohol fuels for internal combustion engines, furnaces, boilers, kilns and gasifiers. United States Patent US 7559961 B2. 2009.

[33] Palmqvist E, Hahn-Hägerdal B (2000). Fermentation of lignocellulosic hydrolysates. II: inhibitors and mechanisms of inhibition. Bioresour Technol.

[34] Kurokawa M, Seno S, Matsuda H, Ying B-W (2016). Correlation between genome reduction and bacterial growth. DNA Res.

[35] Goers L, Freemont P, Polizzi KM (2014). Co-culture systems and technologies: taking synthetic biology to the next level. J R Soc Interface.

