# Supplementary material for: Bioconversion of distillers’ grains hydrolysates to advanced biofuels by an Escherichia coli co-culture
Source: Microb Cell Fact. 2017 Nov 9;16:192. doi: 10.1186/s12934-017-0804-8 (PMC5679325; doi:10.1186/s12934-017-0804-8)
Supplement: Supplementary file 1 — Additional file 1. Additional figures and table. [file 12934_2017_804_MOESM1_ESM.docx]

**Supplementary Materials**

a


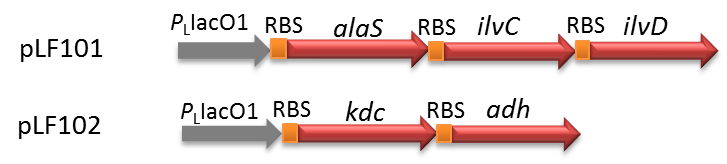


b


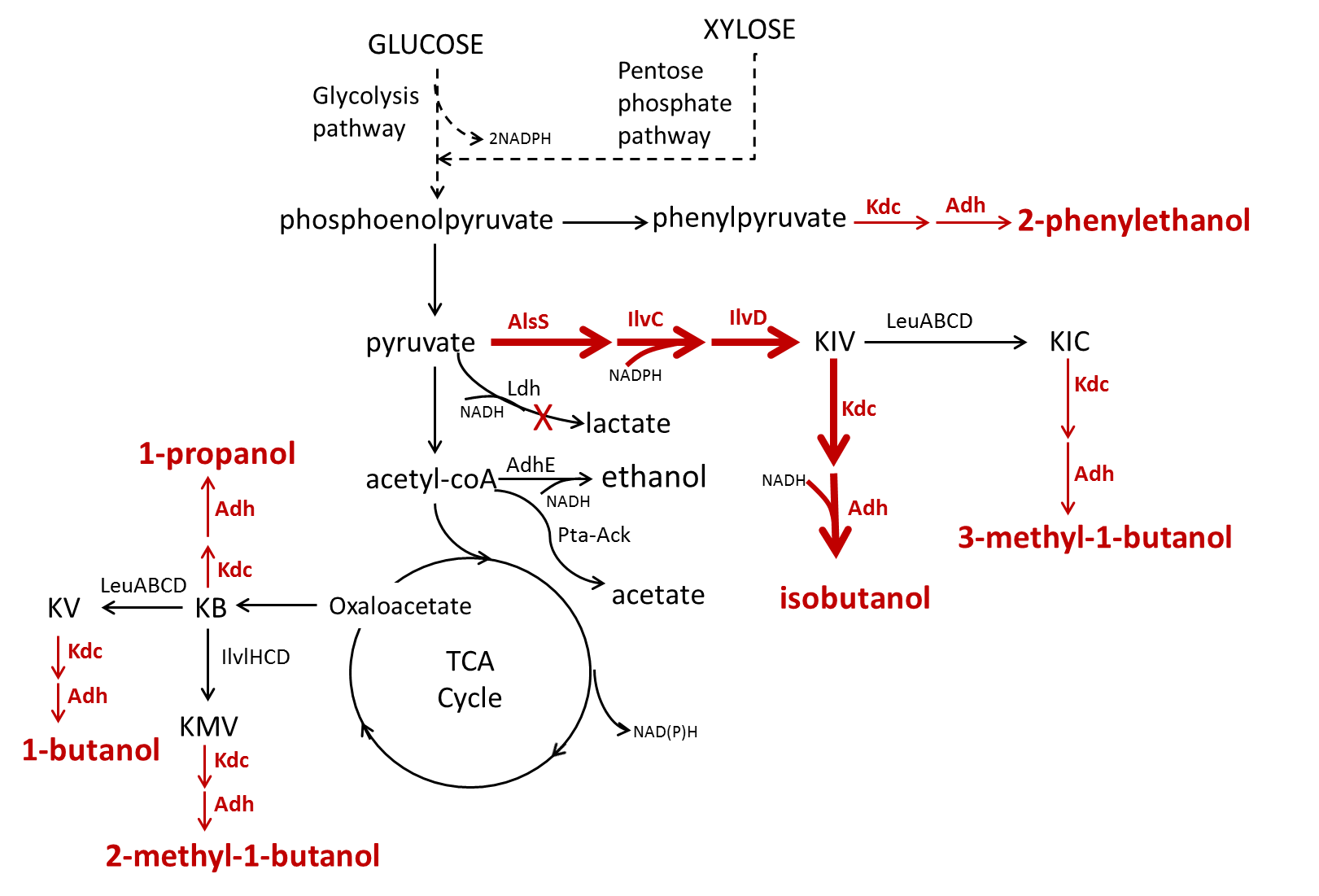


**Figure S1.** The 2-keto acid pathways for fusel alcohol production in *E. coli*. **a** The two constructed plasmids containing the genes encoding for the five enzymes involved in the isobutanol production pathway. **b** The 2-keto acid pathways for fusel alcohol production from glucose and xylose in *E. coli* BLF2. The five genes overexpressed and the corresponding higher fusel alcohol products are marked in red color. AlsS (acetolactase synthase), IlvC (acetohydroxy acid isomeroreductase), IlvD (dihydroxy-acid dehydratase), Kdc (2-ketoacid decarboxylase), Adh (alcohol dehydrogenase), Ldh (lactate dehydrogenase), Pta (phosphotransacetylase), Ack (acetate kinase), KIV (2-ketoisocaproate), KIC (2-ketoisocaproate), KV (2-ketovalerate), KB (2-ketobutyrate), KMV (2-keto-3-methyl-valerate).


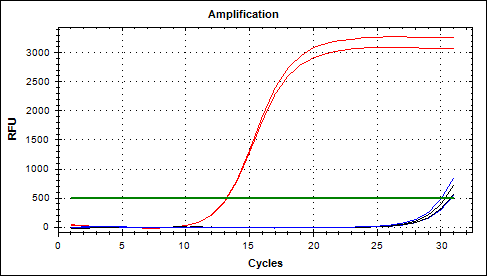

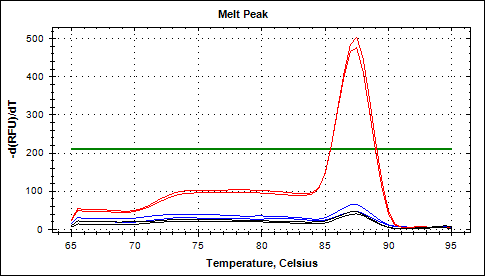


d

c

b

a

NC

AY3

BLF2

NC

AY3

BLF2


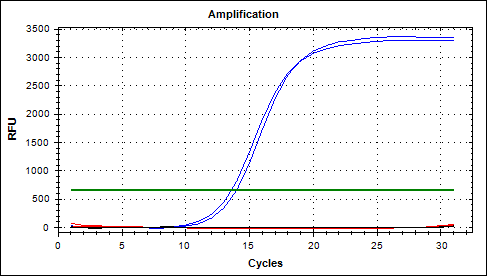

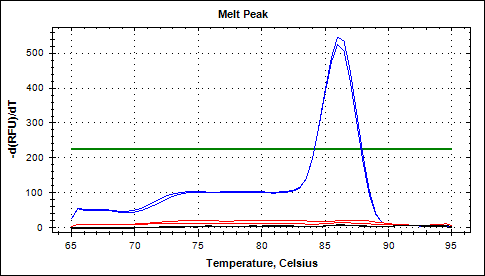


AY3

NC

NC

BLF2

AY3

BLF2

**Figure S2.** Quantitative PCR reactions for the amplification of BLF2 and AY3 genomic DNA and corresponding melt-curve analysis of the specificity of the reactions. The red curves are the PCR reactions using BLF2 genomic DNA as templates, and the blue curves are the PCR reactions using AY3 genomic DNA as templates. NC is negative control using D.I. water as template. **a** q-PCR reactions using primers specific to the *araE* gene of the BLF2 strain. **b** Melting curve analysis of the q-PCR reactions in a. The single peak indicates the absence of unspecific products. **c** q-PCR reactions using primers specific to *malB* gene of the AY3 strain. **d** Melting curve analysis of q-PCR reactions in c.


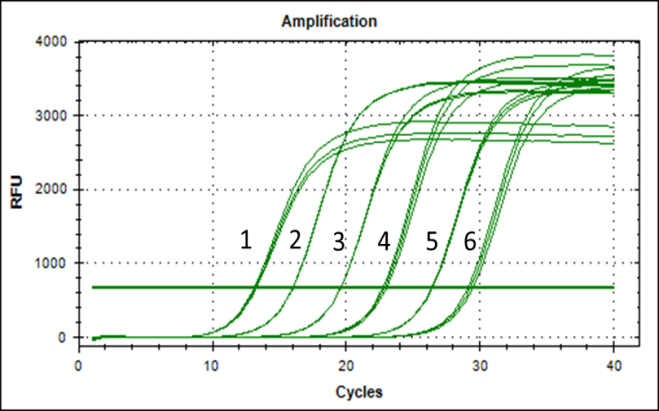

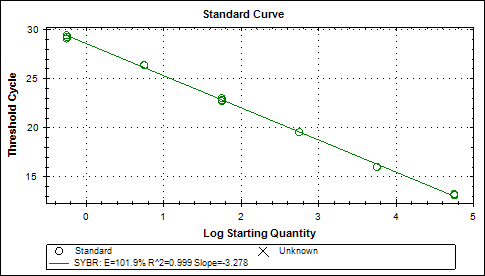


c

d

a

b


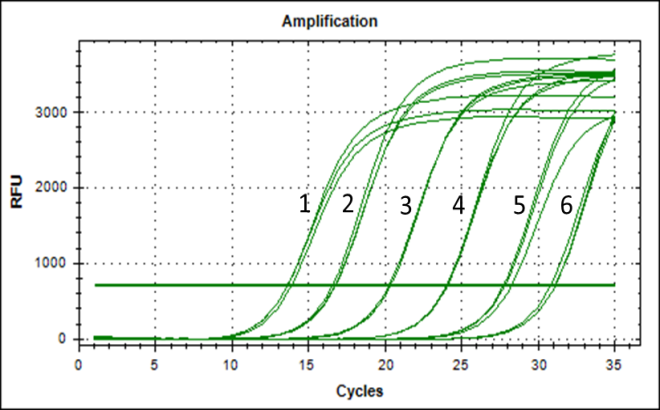

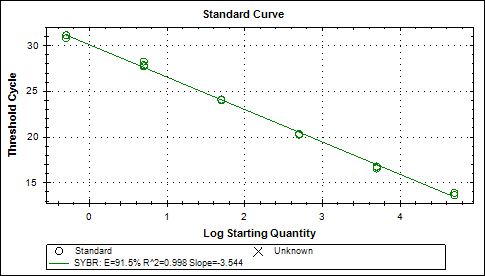


**Figure S3.** Quantitative PCR efficiency test. **a** q-PCR of 1:10 series dilution of the BLF2 genomic DNA samples. **b** The plot of C_T_ for each 1:10 dilution as a function of the log of the starting quantity of BLF2 genomic DNA. The slope of line represents the q-PCR efficiency E of BLF2. **c** The plot of C_T_ for each 1:10 dilution as a function of the log of the starting quantity of AY3 genomic DNA. The slope of line represents the q-PCR efficiency E of AY3.

| Target | *E* | *C_T, R_* | *CFU_R_* | *V_R_* |
| --- | --- | --- | --- | --- |
| BLF2 | 1.019 | 14.79 | 1.09×10^9^ | 2 |
| AY3 | 0.915 | 15.39 | 6.19×10^8^ | 2 |

**Table S1** **Parameters for calculating the cell number of BLF2 and AY3 in the co-culture by the equation in the Methods section**
